# Relationship between the uptake of ^18^F-borono-L-phenylalanine and L-[methyl-^11^C] methionine in head and neck tumors and normal organs

**DOI:** 10.1186/s13014-017-0763-6

**Published:** 2017-01-14

**Authors:** Yoshiaki Watanabe, Hiroaki Kurihara, Jun Itami, Ryohei Sasaki, Yasuaki Arai, Kazuro Sugimura

**Affiliations:** 1 Department of Radiology, Kobe University Graduate School of Medicine, Kobe, Japan; 2 Department of Diagnostic Radiology, National Cancer Center Hospital, 5-1-1 Tsukiji, Chuo-ku, Tokyo, 104-0045 Japan; 3 Department of Radiation Oncology, National Cancer Center Hospital, Tokyo, Japan; 4 Division of Radiation Oncology, Kobe University Graduate School of Medicine, Hyogo, Japan

## Abstract

**Background and purpose:**

The purpose of this study was to determine the distribution of 4-borono-2-^18^F-fluoro-phenylalanine (^18^F-BPA) and L-[methyl-^11^C] methionine (^11^C-Met) in normal organs and tumors and to evaluate the usefulness of ^11^C-Met/PET in screening potential candidates for boron neutron capture therapy (BNCT).

**Material methods:**

Seven patients who had at least one histologically confirmed head and neck tumor were included in this study. They underwent both whole-body ^18^F-BPA-PET/CT and ^11^C-Met-PET/CT within a span of 6 months. Uptake was evaluated using the maximum standardized uptake value (SUVmax). Regions of interest (ROIs) were placed within the tumors and target organs of brain, thyroid, submandibular gland, lung, liver, esophagus, stomach pancreas, spleen, muscle, and bone marrow.

**Results:**

The tumor SUVmax of FBPA and ^11^C-Met showed strong correlation (*r* ^2^ = 0.72, *P* = 0.015). Although ^18^F-BPA and ^11^C-Met showed markedly different uptake in some organs (submandibular gland, liver, heart, stomach pancreas, spleen, and bone marrow), the uptake of ^11^C-Met was consistently higher than that of ^18^F-BPA in these cases.

**Conclusion:**

^11^C-Met PET/CT might be used instead of ^18^F-BPA PET/CT to predict the accumulation of ^10^B in tumors and to select candidates for BNCT. However, it would not be suitable for evaluating accumulation in some normal organs. Therefore, the ^18^F-BPA-PET study remains a prerequisite for BNCT. This is the first report of the correlation between ^18^F-BPA and ^11^C-Met accumulation.

## Background

Boron neutron capture therapy (BNCT) has recently attracted attention and has been used for brain tumors, head and neck cancers, and melanoma [1–4]. It is a targeted radiotherapy method, based on the nuclear reaction of neutrons and ^10^B. After the injection of a ^10^B carrier, it accumulates in target tumor cells. The region to be treated is then exposed to thermal neutrons, and the nuclear reaction of these neutrons with ^10^B produces alpha particles and ^7^Li at very short range (<10 μm), causing lethal damage to tumor cells. The success of BNCT is dependent on the sufficient accumulation of ^10^B in tumor cells and the minimization of such accumulation in normal cells [1, 2, 5]. ^10^B accumulation is not consistent across cases, and is reported to depend upon tumor type. However, even tumors of the same grade may differ in terms of their biochemical properties. Therefore, it is necessary to assess ^10^B accumulation in each case prior to performing BNCT [6].

^10^B-borono-L-phenylalanine (BPA) is the most frequently used ^10^B carrier, and ^18^F-BPA, an ^18^F-labelled analog of BPA, has been developed to predict ^10^B accumulation in tumors and normal tissues by positron emission tomography (PET) [7, 8]. Before BNCT, a ^18^F-BPA-PET study is carried out to evaluate the tumor-to–normal tissue accumulation ratio (TNR). The TNR strongly influences the success of BNCT, and the ideal TNR has been reported to be greater than 2.5 to 5 [1, 5]. However, the synthesis of ^18^F-BPA, a radiolabeled amino acid, is limited due to low radio yield, low synthetic yield, and high cost. Therefore few hospitals or institutions can synthesize ^18^F-BPA in practice [9].

^11^C -labelled Methionine (^11^C-Met) is the most popular radiolabeled amino acid. Methionine is an essential amino acid and plays an important role in protein synthesis as it is coded for by the initiation codon. Radiolabeled methionine is a convenient radiochemical product because it can be obtained by rapid synthesis with high radiochemical yield [10]. ^11^C-Met is a highly sensitive tool capable of yielding considerable information on protein synthesis, and has been used widely in the assessment of various cancers [11, 12]. Compared to ^18^F-fluorodeoxyglucose, ^11^C-Met shows low uptake in the brain, so ^11^C-Met PET can evaluate head and neck tumors without interference from physiological accumulation in the brain [13, 14].

In this study, we evaluated the distribution of these two radiolabeled amino acids, ^18^F-BPA and ^11^C-Met, in patients with head and neck tumors, in order to evaluate whether ^11^C-Met PET can be used instead of ^18^F-BPA PET for screening potential candidates for BNCT.

## Materials and methods

### General

All chemical reagents were obtained from commercial sources. This study was conducted according to a protocol approved by the institutional review board/independent ethics committee of the National Cancer Center Hospital (Tokyo, Japan). All patients signed a written informed consent form before the initiation of the study.

### Radiosynthesis of ^18^F-BPA and ^11^C-MET

^18^F-BPA was synthesized by direct electrophilic radiofluorination of BPA (Sigma-Aldrich, St. Louis, MO, USA) using ^18^F-acetyl hypofluorite as described previously [6]. Purification of ^18^F-BPA was performed by high performance liquid chromatography (HPLC) using a YMC-Pack ODS-A column (20 × 150 mm; YMC, Kyoto, Japan) eluted with 0.1% acetic acid at a flow rate of 10 ml/minute. The radiochemical purity of ^18^F-BPA as determined by HPLC was > 99.5%, while its specific activity was 25 MBq/μmol.

^11^C-Met was synthesized in the radiochemical laboratory of this institute by methylation with L-homocysteine thiolactone followed by isolation of the final product by solid phase extraction using L-homocysteine thiolactone (Sigma-Aldrich, St. Louis, MO, USA) [10]. The radiochemical purity of ^11^C-Met ranged from 95 to 98%.

### Subjects

The seven patients included in this study underwent both ^18^F-BPA-PET/computed tomography (CT) and ^11^C-Met-PET/CT at least 24 h apart, but within 6 months of each other, between January 2014 and July 2016. Of the 116 patients who underwent ^18^F-BPA PET/CT, seven also underwent ^11^C-Met-PET/CT. These seven patients were retrospectively selected for this study. They had histologically confirmed head and neck tumors, Eastern Cooperative Oncology Group performance status (PS) of 0–1, adequate organ function (neutrophil count ≥ 1500 /μL, platelet count ≥ 75,000 /μL, hemoglobin concentration ≥ 9.0 g/dL, serum bilirubin ≤1.5 mg/dL, aspartate transaminase (AST) and alanine aminotransferase (ALT) ≤ 100 IU/L, serum creatinine ≤ 1.5 mg/dL, baseline left ventricular ejection fraction (LVEF) > 60%), and were older than age 20. For evaluating the distribution of ^11^C-Met and ^18^F-BPA in normal organs, we excluded patients with congestive heart failure, uncontrolled angina pectoris, arrhythmia, symptomatic infectious disease, severe bleeding, pulmonary fibrosis, obstructive bowel disease or severe diarrhea, and symptomatic peripheral or cardiac effusion.

Patients fasted for at least 4 h before examination.

### PET/CT protocol

PET/CT images were acquired with a Discovery 600 scanner (GE Healthcare, Milwaukee, WI, USA). Whole-body ^18^F-BPA PET/CT imaging was carried out at 1 h after the injection of ^18^F-BPA (ca. 4 MBq/kg). The scan timing for ^18^F-BPA PET/CT was determined as in our previous work [6]. Whole-body ^11^C-Met PET/CT was carried out 10 min after the injection of ^11^C-Met (ca. 4 MBq/kg). The scan timing for^11^C-Met PET/CT was determined by a previous report [15]. A scout image was first acquired to determine the scanning field range from the head to the pelvis of the patient, using settings of 10 mA and 120 kV. Next, whole-body 16-slice helical CT and whole-body 3D PET were performed. PET images were acquired in 7–8 bed positions with 2-min acquisition durations per bed position, such that the images covered the same field as the whole-body CT image. The acquired data were reconstructed as 192 × 192 matrix images (3.65 × 3.65 mm) using a 3D ordered subsets-expectation maximization algorithm.

### Tumor uptake of ^18^F-BPA and ^11^C-Met

PET image evaluation and quantification of the standardized uptake value (SUV) were performed using AW Volume Share 4.5 software (GE Healthcare, Milwaukee, WI, USA). Regions of interest (ROIs) were delineated on the axial 2-D image. SUV was defined as regional radioactivity divided by injected radioactivity normalized to body weight. ^18^F-BPA uptake was evaluated using the maximum SUV (SUVmax) 1 h after injection, while ^11^C-Met uptake was evaluated 10 min after injection. ROIs were placed within the tumor and target organs of brain (white matter), thyroid, submandibular gland, lung, liver, esophagus (cervical esophagus), stomach, pancreas, spleen, muscle (latissimus dorsi), and bone marrow (12th thoracic vertebra). Tumor ROIs were defined as the areas of highest activity. ROIs were also placed onto normal tissue surrounding the tumor to calculate the TNR of ^18^F-BPA. Clinically, dose planning is performed based on the TNR prior to the initiation of BNCT to avoid severe damage to normal tissues.

### Statistical analysis

For statistical analysis of the data, JMP software (version 9.0, SAS Institute, Inc., Cary, NC) was used. A linear regression analysis was performed for the correlation study. Fisher’s exact test was used to estimate the concordance of the cut-off values of the two tracers. Probability values of *P* < 0.05 were considered significant. Since patients in previous studies were determined to be eligible for BNCT when the TNR of ^18^F-BPA was more than 2.5 [1, 6, 8, 16], we used a ^18^F-BPA TNR of more than 2.5 as a cut-off to distinguish positive from negative.

## Results

Seven patients with head and neck tumors underwent both ^18^F-BPA-PET/CT and ^11^C-Met-PET/CT during the study period and were enrolled in this study (six males and one female; ages 20 to 66 years, median 47 years).

Patient and tumor characteristics are summarized in Table 1. In terms of the primary disease type, two patients had facial rhabdomyosarcoma, one had external auditory canal cancer, one had lingual cancer, one had malignant melanoma of the nasal cavity, one had parotid gland cancer, and one had adenoid cystic carcinoma of the lacrimal sac. Histological diagnoses included two patients with squamous cell carcinoma (SCC), two with adenoid cystic carcinoma (ACC), two with rhabdomyosarcoma (RBD), and one with malignant melanoma. Four patients experienced local recurrence (LR), one had distant metastases, and two had a newly diagnosed second malignancy. All patients had unresectable tumors. All lesions were located within the head and neck region (two lesions in the maxillary sinus, one in the external auditory canal, one in the nasal cavity, one in the tongue, one in the parotid gland, and one in the orbit). Tumor size scaled by PET/CT ranged from 2 to 5 cm. The tumor size did not change in the interval between PET scans in six cases, but in one case (No. 1) the tumor grew from 3 cm to 5 cm. Five patients underwent surgery and received radiotherapy and chemotherapy within 1 year before the PET scans, while two patients did not receive any treatment before the scans. Neither chemotherapy nor radiation therapy was performed in any patient in the interval between PET scans, but one patient (No. 1) underwent palliative surgery during this time. However, in case No. 1 the target tumor was almost unresectable and we were able to evaluate radioisotope accumulation in the tumor tissue. In four cases, ^18^F-BPA-PET/CT was performed prior to ^11^C-Met-PET/CT. In the remaining three cases, ^18^F-BPA-PET/CT was performed after ^11^C-Met-PET/CT. The interval between studies was less than 3 weeks in six cases, and more than 3 months in one case (No. 1). The interval ranged from 2 to 123 days (mean, 23 ± 41; median, 5).

**Table 1:** Patient and tumor characteristics

No.	Sex	Age	Primary disease	Presentation	Histology	Location	Past therapy	Size (cm)	Interval between studies (days)	Tumor SUVmax		Tumor TNR	
										^11^C-Met	^18^F-BPA	^11^C-Met	^18^F-BPA
1	M	20	Facial RBD	NT	RBD	orbit	No^a^	3.2 × 3.0 to 4.5 × 5.0^b^	123	3.7	3.6	3.8	3.9
2	M	20	Facial RBD	LR	RBD	MS	PO/CRT	2.5 × 1.5	5	1.5	1.6	1.1	1.3
3	M	27	Lingual	LR	SCC	Lingual	PO/CRT	3.2 × 4.1	16	6.6	4.2	4.6	3.1
4	M	50	EAC cancer	LR	SCC	EAC	PO /CRT	3.3 × 4.4	2	4.4	4.1	3.1	2.9
5	M	47	Parotid gland ACC	LR	ACC	Parotid gland	PO /CRT	2.0 × 2.3	2	5.6	5.6	5.1	2.7
6	M	47	Nasal melanoma	NT	Melanoma	Nasal cavity	No	3.1 × 3.2	4	5.8	4.7	4.1	3.9
7	F	66	Lacrimal ACC	M	ACC	MS	PO /CRT	3.3 × 2.2	6	4.7	3.6	2	2.2
								Average	5 (median)	4.6 ± 1.7 (mean)	3.9 ± 1.2 (mean)	3.4 ± 1.4 (mean)	2.9 ± 0.9 (mean)

*RBD* rhabdomyosarcoma, *EAC* external auditory canal, *ACC* adenoid cystic carcinoma, *NT* newly diagnosed tumor, *LR* local recurrence, *M* Metastasis, *SCC* squamous cell carcinoma, *MS* maxillary sinus, *PO* postoperative, *CRT* chemoradiation therapy

^a^Palliative surgery was performed in the interval between PET scans. ^b^Tumor size increased in the interval between PET scans

**Fig. 1:**
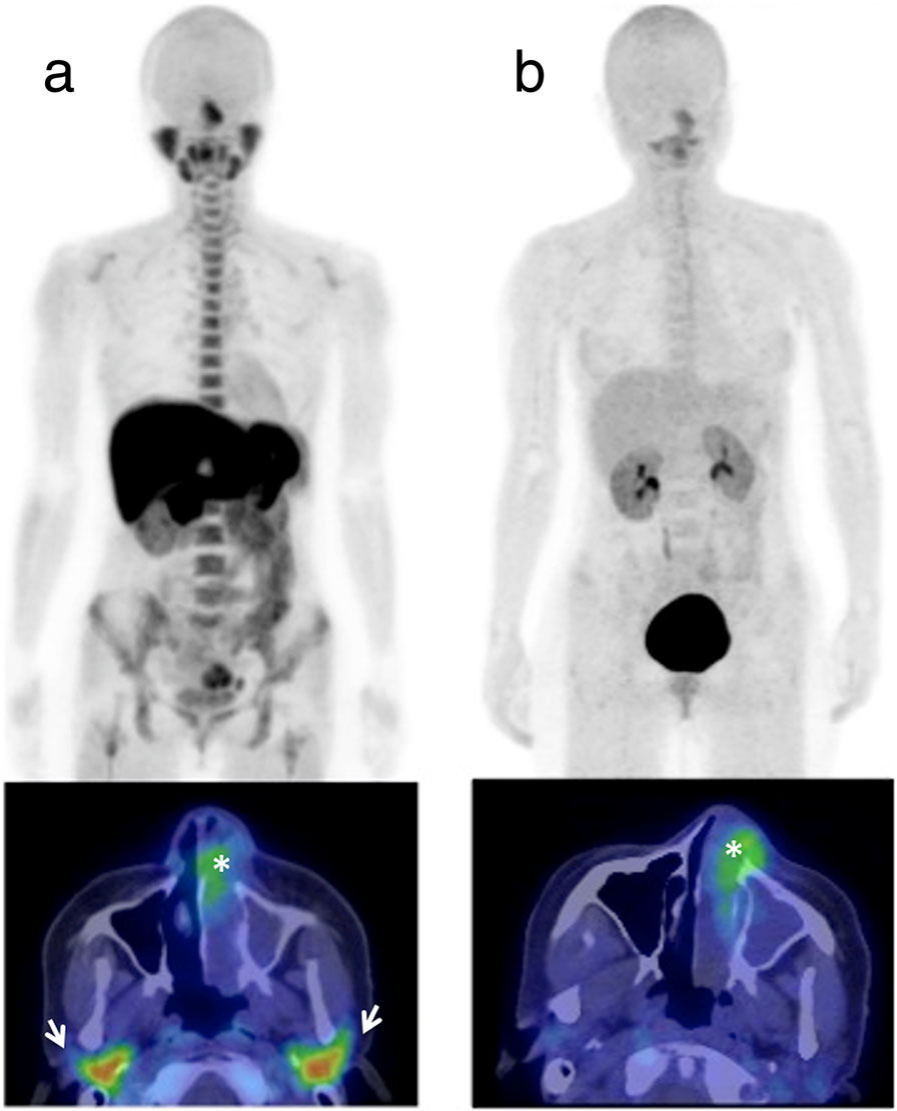
Representative ^11^C-Met and ^18^F-BPA PET/CT (patient No. 6). *Upper panel*: maximum intensity projection imaging. *Lower panel*: PET/CT fusion image. **a** ^11^C-Met-PET/CT at 10 min after injection. Tumor SUVmax was 5.8. (*). High physiological uptake is shown in the salivary glands (*arrows*), bone marrow, and some abdominal organs. **b** ^18^F-BPA-PET/CT at 1 h after injection. Tumor SUVmax was 4.7. (*). Physiological uptake is generally low

Representative PET/CT imaging of ^18^F-BPA and ^11^C-Met is shown in Fig. 1. SUVmax values of ^18^F-BPA and ^11^C-Met in tumor tissue are summarized in Table 1. The accumulations of ^18^F-BPA and ^11^C-Met varied widely among tumor cases, even in those with the same pathology. The tumor SUVmax of ^18^F-BPA ranged from 1.6 to 5.6 (mean, 3.9 ± 1.4), while that of ^11^C-Met ranged from 1.5 to 5.8 (mean, 4.6 ± 1.7). The TNRs of both ^18^F-BPA and ^11^C-Met also varied widely. The TNR of ^18^F-BPA ranged from 1.3 to 3.9 (mean, 2.9 ± 0.9), while that of ^11^C-Met ranged from 1.1 to 5.1 (mean, 3.4 ± 1.4).

**Fig. 2:**
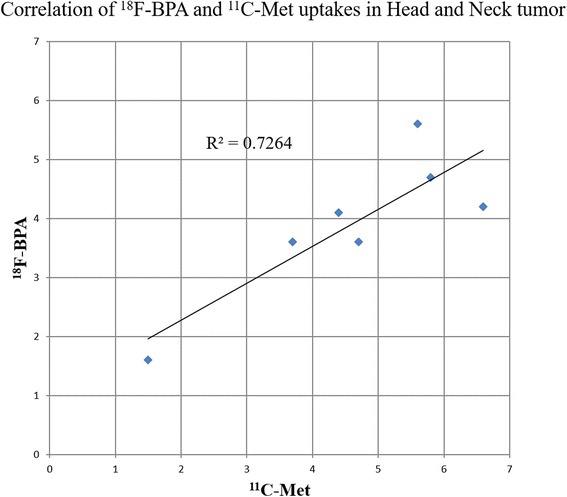
Correlation of SUVmax between ^18^F-BPA and ^11^C-Met in head and neck tumors. A close linear correlation is observed between FBPA uptake and MET uptake in head and neck tumors (*r* ^2^ = 0.72, *P* = 0.15)

The TNRs of ^18^F-BPA and ^11^C-Met were weakly correlated (*r* ^2^ = 0.51), though statistical significance was not observed (*P* = 0.07). However, the SUVmax of ^18^F-BPA and ^11^C-Met within each tumor exhibited strong correlation (Fig. 2; *r* ^2^ = 0.72, *P* = 0.015).

Accumulations of ^11^C-Met and ^18^F-BPA in normal organs are summarized in Table 2. As expected, accumulations of ^18^F-BPA in normal organs showed smaller differences between patients than accumulations in tumors.

**Table 2:** Average and standard deviation (SD) of ^18^F-BPA and ^11^C-Met uptake in normal organs

	^11^C-Met	^18^F-BPA	*P* value
Brain	1.5 ± 0.1	1.3 ± 0.3	0.31
Submandibular gland	5.2 ± 1.2	2.0 ± 0.5	<<0.01
Thyroid gland	2.1 ± 0.4	1.4 ± 0.5	0.7
Esophagus	2.3 ± 0.6	1.9 ± 0.6	0.18
Lung	0.7 ± 0.1	0.6 ± 0.2	0.76
Heart	2.5 ± 0.4	1.2 ± 0.1	<<0.01
Blood	1.5 ± 0.2	1.3 ± 0.1	0.07
Stomach	8.8 ± 2.6	1.5 ± 0.2	<<0.01
Liver	11 ± 1.8	2.0 ± 0.5	<<0.01
Pancreas	12.6 ± 2.8	1.7 ± 0.3	<<0.01
Spleen	3.1 ± 0.6	1.5 ± 0.3	<<0.01
Kidney	4.0 ± 0.4	3.4 ± 0.9	0.18
Muscle	1.3 ± 0.1	1.2 ± 0.2	0.21
Bone marrow	3.8 ± 0.8	1.5 ± 0.3	<<0.01
Tumor	4.6 ± 1.7	3.9 ± 1.2	0.39

Across all patients, the uptake of ^18^F-BPA and ^11^C-Met differed widely in the submandibular gland, liver, heart, stomach, pancreas, spleen, and bone marrow. In these organs, the uptake of ^11^C-Met was consistently higher than that of ^18^F-BPA. In all other organs, no significant difference was observed.

## Discussion

It is well-known that accumulation of radioisotopes is affected by nature of tumors [17, 18]. In most cases in this study, PET/CTs were performed as preparation for therapy and the intervals between scans were less than 3 weeks. However, in one case (No. 1), the interval between ^18^F-BPA-PET/CT and ^11^C-Met-PET/CT was more than 3 months; palliative surgery was performed during the interscan interval, although the target tumor was almost unresectable. The long interval and palliative operation may have affected the results in this case. However, excluding the single long-interval case, the TNRs of ^18^F-BPA and ^11^C-Met showed a weak correlation (*r* ^2^ = 0.57 *P* = 0.08), and the SUVmax of ^18^F-BPA and ^11^C-Met within each tumor were strongly correlated (*r* ^2^ = 0.73, *P* = 0.03). As a result, we do not believe that the interscan interval or the palliative operation particularly influenced the results of this study. On the other hand, five patients underwent surgery and received radiotherapy and chemotherapy within 1 year before the PET scans, and it is unclear whether this treatment influenced our results.

Radiolabeled amino acids are among the most important tracers for identifying and examining tumors, since cellular proliferation requires protein synthesis. Amino acids are the natural building blocks of proteins, and high uptake of these precursors is a normal feature of rapidly proliferating cells such as tumor cells. Tumor cells take up amino acids by amino acid transporters, thus the numbers of such transporters is increased in most tumor types as compared to healthy tissue [19]. Studies have shown that there are a variety of amino acid transporters, such as System L, System A, System ASC, and System B [9, 20], and ^18^F-BPA is a System L–specific imaging agent [9]. System L is Na^+^-independent and is a major nutrient transport system responsible for the transport of neutral amino acids. System L includes four families, LAT1–LAT4.

^18^F-BPA uptake correlates with total LAT expression, but more specifically with that of LAT1 and LAT4, which are overexpressed in many tumors [9, 21–23]. On the other hand, ^11^C-Met is taken up not only by System L, but also by many other types of amino acid transporter such as System A, System ASC, and System B [20, 24, 25]. It has been previously reported that the expression of amino acid transporters in tumors varies widely, and it sometimes reflects proliferation speed and malignancy [26]. This may explain the wide variation in the tumor accumulation of ^18^F-BPA and ^11^C-Met in this study, regardless of pathology. Despite the fact that ^11^C-Met and ^18^F-BPA are affected by different amino acid transporters, we found that ^11^C-Met uptake correlated closely with ^18^F-BPA uptake. One possible explanation is that the rates of amino acid transport and protein synthesis are so rapid that differences in the types of amino acid transporter may not be significant. Further studies with larger numbers of participants and comparison of particular histological features should be performed to resolve this question.

In normal organs, we found that accumulation differed between ^11^C-Met and ^18^F-BPA in the submandibular gland, liver, heart, stomach, pancreas, spleen, and bone marrow, whereas it was similar in all other normal organs. Previous papers have also reported similar tendencies in ^11^C-Met distribution [15] . The uptake of ^11^C-Met and ^18^F-BPA in normal organs showed smaller individual differences than in tumors. The variation in relative accumulation between tumors and healthy organs may be attributed to the histological heterogeneity of tumors. Similarly, there was no significant difference between accumulations in brain, thyroid, lung, esophagus, and muscle. These organs had insufficient accumulations of both ^11^C-Met and ^18^F-BPA to reveal any difference, perhaps because there is less protein synthesis and cell proliferation in these organs, making the uptake of the amino acids themselves very low.

On the other hand, some normal organs showed higher uptake of ^11^C-Met than of ^18^F-BPA. This difference was especially pronounced in organs with high levels of protein synthesis or cell proliferation, such as the submandibular gland, pancreas, liver, stomach, and bone marrow. The heart produces several hormones, such as brain natriuretic peptide. The spleen sometimes has extramedullary hematopoietic functions in cases where the bone marrow has been damaged. Thus both organs can be considered to be involved in protein synthesis. Although these organs all require amino acids to synthesize proteins, the pancreas, liver, stomach, heart, and submandibular gland do not express LAT1 or LAT4, while the bone marrow and spleen express LAT1 but not LAT4 [27, 28]. These latter organs may exhibit less ^18^F-BPA uptake because is transported mainly by LAT1 and LAT4 [9]. As mentioned, ^11^C-Met can be transported by many types of amino acid transporter [9, 20], including System A, expressed by the stomach, liver, pancreas, heart, and spleen [29], and System B (^0,+^), expressed by the salivary glands [30]. This could explain the fact that the accumulation of ^11^C-Met in these organs was higher than that of ^18^F-BPA, and it may be that variation in the distribution of amino acid transporters causes differences in uptake. In other words, the greater accumulation of ^11^C-Met may suggest that its uptake reflects protein synthesis more generally, whereas the uptake of ^18^F-BPA also reflects the expression of LAT1 and LAT4.

Our study also revealed a low physiological accumulation of ^18^F-BPA in normal organs. This may be of great advantage, not only in the evaluation of ^10^B accumulation, but in tumor detection. It could be especially useful when evaluating tumors in the liver, the pancreas, or stomach. In particular, a study on bile duct carcinoma, including pancreatic cancer, found that the expression of LAT 1 correlated positively with degree of malignancy [26], thus the accumulation of ^18^F-BPA might predict the malignancy of these cancers.

Our findings suggest that the accumulation of ^11^C-Met may predict the accumulation of ^18^F-BPA in tumors. Nevertheless, for success of BNCT, performance of the TNR is more important than evaluation of SUVmax [1, 5].

In all cases where the tumor TNR of ^11^C-Met was positive, the tumor TNR of ^18^F-BPA was also positive. However, the correlation between the TNRs of ^18^F-BPA and ^11^C-Met was not statistically significant. It is inferred that the accumulation in normal tissues surrounding the tumor differed between ^18^F-BPA and ^11^C-Met. In this study, as the organs demonstrating high uptake of ^11^C-Met were located far from the tumors, or post resection, we were able to a certain extent to evaluate the TNR by ^11^C-Met PET/CT. However, if a tumor were near such organs, it would disturb the evaluation of the TNR.

## Conclusion

Despite variations in tumor pathology and a small patient population, ^18^F-BPA accumulation in tumors showed a strong correlation with ^11^C-Met accumulation. Thus, ^11^C-Met PET/CT might be useful to select candidates for ^18^F-BPA PET/CT. However, ^11^C-Met PET/CT would not be suitable for evaluating accumulation in some normal organs, such as the submandibular gland, liver, heart, stomach pancreas, spleen, and bone marrow. Therefore, the ^18^F-BPA-PET study remains a prerequisite for BNCT. However, further studies are called for, using larger numbers of participants and selection of particular histological diagnostic criteria.

### Limitations

As mentioned, some limitations should be acknowledged. First, the very small population and retrospective nature of this study may have led to selection bias. Second, the study included a variety of tumor pathologies.

## Abbreviations

^11^C-Met: L-[methyl-^11^C] methionine
ALT: Alanine aminotransferase
AST: Aspartate transaminase
BNCT: Boron neutron capture therapy
BPA: Borono-L-phenylalanine
CT: Computed tomography
HPLC: High-performance liquid chromatography
LAT: System L amino acid transporter
LVEF: Baseline left ventricular ejection fraction
PET: Positron emission tomography
PS: Eastern Cooperative Oncology Group performance status
ROI: Region of interest
SUVmax: The maximum standardized uptake value
TNR: Tumor-to–normal tissue accumulation ratio
